# Midwives’ and Nurse Midwives’ Autonomy Experience in Caring for Laboring Women in Health Facilities: A Systematic Review With Metasynthesis

**DOI:** 10.1177/23779608261433120

**Published:** 2026-09-07

**Authors:** Elsa Salgueiro, Catarina Afonso, Margarida Sim-Sim, Claúdia Janeiro, Maria Otília Zangão

**Affiliations:** 1 Nova University Lisbon, 70861 Comprehensive Health Research Center, Nova National School of Public Health, Lisbon, Portugal; 2 70989 University of Évora, Comprehensive Health Research Centre, Évora, Portugal; 3 Alto Alentejo Local Health Unit, Dr. José Maria Grande Hospital, Portalegre, Portugal; 4 Polytechnic Institute of Santarém, 112018 RISE-Health Integrated Member, Higher School of Health, Santarém, Portugal; 5 70989 University of Évora, Comprehensive Health Research Centre, São João de Deus Higher Nursing School, Évora, Portugal

**Keywords:** labor, life change events, midwifery, nurse midwives, professional autonomy, systematic review

## Abstract

**Introduction:**

The World Health Organization emphasizes the autonomy of Midwives and Nurse Midwives in physiological care during childbirth. Despite international regulation of competencies, the expression of autonomy isn’t consistent, potentially creating inconsistencies in clinical care and professional identity. The objective was to synthesize evidence on the experiences of Midwives and Nurse Midwives regarding autonomy in caring for laboring women in health facilities.

**Methods:**

Systematic review with metasynthesis, according to the methodology of the Joanna Briggs Institute, carried out by meta-aggregation. Nine platforms and databases were searched for primary studies published from 2016/2024 in English, French, Spanish, and Portuguese. The selection of studies followed the PRISMA guidelines. Sixteen articles met the eligibility and methodological quality criteria. Registered protocol in PROSPERO.

**Results:**

Three categories emerged: (a) autonomy as a context of safety and confidence, (b) autonomy as a process of strengthening identity, and (c) autonomy as a reflection of expertise. These generated the meta-category: The dynamics of Midwives/Nurse Midwives’ autonomy.

**Conclusion:**

The autonomy of Midwives and Nurse Midwives in “being and doing” affirms their identity, expresses their expertise, and promotes the confidence and safety of laboring women. Autonomous Midwives and Nurse Midwives make childbirth a unique and unrepeatable event while defending physiological care. Recognizing the autonomy of Midwives and Nurse Midwives strengthens global health.

## Introduction

Professional autonomy is a key challenge for many Midwives and Nurse Midwives (M/NM) who are part of the wider midwifery workforce (UNFPA, ICM, WHO, 2021). M/NM are qualified and experienced professionals who possess specialized knowledge and skills for which they are responsible and accountable (UNFPA, ICM, WHO, 2021; Vermeulen, Fobelets, et al., 2023). This professional foundation allows them to make independent decisions and work autonomously (Clemons et al., 2021; Vermeulen, Fobelets, et al., 2023).

The expert consensus identifies three fundamental dimensions of autonomy in midwifery: working conditions, professionalism, and interprofessional relationships (Vermeulen et al., 2022). However, the autonomy of M/NM has been eroded, both by the establishment of a technocratic model and by management, which, while advocating good practices, often prevents the implementation of international guidelines (Sonmezer, 2021).

The exercise of autonomy by M/NM is not in compliance with regulations in many countries. In a hospital setting, their practice is often conditioned by the institutional hierarchy (from which they receive orders and assignments) and by the established biomedical model, with repercussions on respect for the physiology of labor. In clinical practice, decisions are often discussed for cesarean deliveries that could have been assisted by M/NM and progressed to a normal delivery. These M/NM are ignored as caregivers. They are considered subordinates of the medical profession. They are denied their professional identity.

The report “Midwives’ Voices Midwives’ Realities” reveals that the limitation of autonomy is noticeable and consciously recognized, contrary to regulations in several countries. Factors that negatively impact the implementation of the functions inherent to M/NM and the development of autonomy include the diversity of academic training plans/levels (WHO, ICM, WRA, 2016).

The support of regulatory associations, midwife-led models, and woman-centered care motivates M/NM to greater autonomy. This increases professional satisfaction, the quality and safety of care, and the cost-benefit balance in health expenditure (Vermeulen, Buyl, et al., 2023; Vermeulen, Fobelets, et al., 2023; Zolkefli et al., 2020). In the global context, it seems urgent and necessary to recognize the practice of M/NM as a truly autonomous profession. (WHO, ICM, WRA, 2016).

The struggle for midwives’ autonomy is historic and reinforced by milestones. The report Midwives’ Voices, Midwives’ Realities (WHO, 2016) is a milestone, bringing to public discussion, in a systematic and comprehensive manner, the challenges faced by M/NM in exercising autonomy before 2016.

The period from 2016 to 2024 is therefore relevant due to the transformations in the field of maternal health, especially in the consolidation of humanization policies. However, this period lacks methodical information that elucidates, in the form of a systematic review, the theme of professional autonomy of M/NM. Specifically, it lacks information on the experiences of autonomy of M/NM in caring for laboring women in health facilities. This gap limits understanding of the experience of autonomy and its expression in clinical practice, which may affect the development of supportive policies and practice environments. With metasynthesis, it is possible to reveal meanings and interpretive patterns that would be lost in separate analyses. In fact, reinterpretation allowed for a greater understanding of the experience and decisions of these professionals, legitimizing the knowledge discovered as scientific evidence.

Thus, the objective was to synthesize evidence on the experiences of M/NM regarding autonomy in caring for laboring women in health facilities.

## Methods

### Design

This is a systematic review of the literature with metasynthesis, which aims to achieve a broad and deep theoretical understanding (Faria & Camargo, 2022) to optimize the validity of the procedures and results (Sandelowski, 2012). The meta-aggregation guidelines of the Joanna Briggs Institute (JBI) (Lockwood et al., 2015) and the PRISMA checklist (Moher et al., 2015) were followed. Protocol registered with PROSPERO.

### Inclusion and Exclusion Criteria

The eligibility criteria for articles followed the PICo acronym to answer the question “What are the experiences of M/NM regarding autonomy in caring for laboring women in health facilities?”:

- Population: M/NM with higher education and practicing as clinicians. The MeSH terms included were midwives, nurse midwives, obstetric nurses, and advanced practice nursing.
- Phenomenon of Interest: Experiences related to autonomy in assisting women in labor. The terms included were autonomy, professional autonomy, professionalism, professional self-regulation, life change event, life experience, experience, and perception.
- Context: Studies carried out in public and private institutions providing care to laboring women. The terms included were parturition, intrapartum care, childbirth, obstetric delivery, and labor.

Other criteria: primary qualitative studies, in English, French, Spanish, and Portuguese, in full text and published between 2016 and 2024. Interpretive studies based on the experiences of M/NM focused on the autonomy of care for laboring women were included, such as phenomenological, ethnographic, grounded theory, action research, and feminist research designs. Mixed-method studies included only qualitative data.

Exclusion criteria included quantitative methodologies, systematic reviews and meta-analyses, narrative reviews, opinion articles, editorials, comments, notes, letters to the editor, opinions, and gray literature. Studies involving traditional midwives and professionals practicing in homes were excluded (Webler et al., 2023). Studies with a quality score <75% on the JBI tool (Lockwood et al., 2015) were rejected.

### Search Strategy

No records of ongoing or completed reviews were identified (PubMed, PROSPERO, Open Science Framework). Protocol registered with PROSPERO.

In the second phase (October 2024), the PubMed database was searched for primary articles on the topic. Titles, abstracts, and relevant indexed terms were analyzed to develop the search strategy. In the third phase, the search strategy was adapted to each database included.

The search was conducted in December 2024 on the EBSCOhost platform (CINAHL Ultimate, MEDLINE Ultimate, Academic Search Complete) and in five direct access databases (LILACS, PubMed, Wiley, Scopus, and Web of Science). The studies in the analysis corpus were imported into Rayyan^®^ software (Ouzzani et al., 2016).

### Article Selection

**Figure 1.**
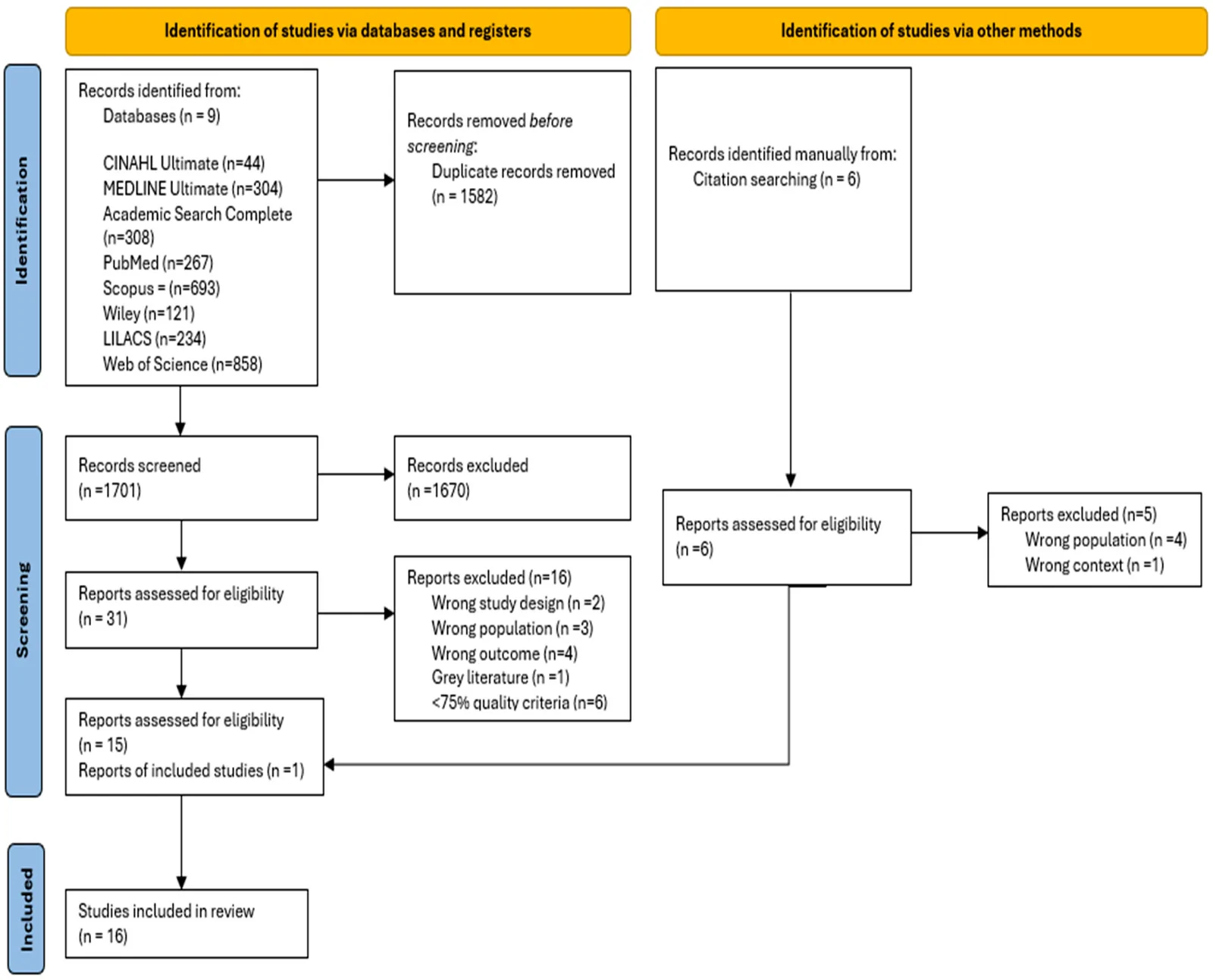
Flowchart for identifying, selecting, and including studies in the metasynthesis (PRISMA).

The study selection process followed the PRISMA flowchart (Moher et al., 2015; Page et al., 2021) (Figure 1). The search yielded 2,929 articles, of which 1,582 duplicates were removed. Two authors independently screened the articles using the titles and abstracts according to the eligibility criteria. A pilot analysis of 25 titles and abstracts was conducted independently by two authors. The results were compared and discussed to refine eligibility.

In the screening of titles and abstracts, 1,670 articles were excluded. Reading the full text of the 31 articles led to the exclusion of ten for not meeting the eligibility criteria and six for not having methodological quality, according to the JBI criteria (Critical Appraisal Checklist for Qualitative Research) (Lockwood et al., 2015). In the selection process, disagreements between reviewers were discussed and consensus was reached.

In the manual scrutiny of the bibliographic reference lists of the selected articles, six studies were identified. Of these, five were excluded because they did not meet the inclusion criteria. The final sample included 16 articles. The selection process took place between January and March 2025.

### Quality Appraisal

The inclusion of the 16 articles was based on the JBI critical appraisal criteria (Lockwood et al., 2015). The articles were evaluated with scores between 80% and 100%. Limitations were found in the researcher's theoretical and cultural location, influence on the research, representation of participants, and their opinions. The studies were evaluated by three authors in April 2025 (Supplemental material—Appendix I. Critical appraisal results of eligible studies)

### Data Extraction

A data extraction tool was constructed, in which three authors recorded the characterizing topics (Supplemental material—Appendix II. Description of Included Articles). Subsequently, four authors independently extracted the conclusions, illustrating them with statements from the primary studies (Supplemental material—Appendix III. Data extraction). Based on this analysis and through collaborative consensus review, the conclusions were grouped into categories according to similarity and significance. This stage was completed in April–May 2025.

### Metasynthesis

For meta-aggregation, the JBI guidelines (Lockwood et al., 2015) were followed. Similarities and differences in participants’ experiences were sought and organized in a cascade, as subcategories, broad categories, and inclusive meta-categories (Supplemental material—Appendix IV. Aggregation process for primary data). By thematic similarity, 67 findings were reached. The contribution of each study to the subthemes varied: some contributed transversally to several subthemes, while others offered specific insights into certain dimensions. For example, Bradfield et al. (2019a, 2019b) contributed significantly to all three themes of the synthesis, while Ferguson et al. (2022) focused on the theme of safety and trust. This variation reflects the diversity of methodological approaches and specific focuses of the primary studies, enriching the final synthesis.

The ConQual approach (Munn et al., 2014) was used to establish the reliability and credibility of the synthesized results. “High” reliability was achieved with a “yes” response to more than four criteria. Credibility was “moderate” when illustrations were assigned unequivocal and credible ratings (Supplemental material—Appendix V. ConQual summary of findings)

**Figure 2.**
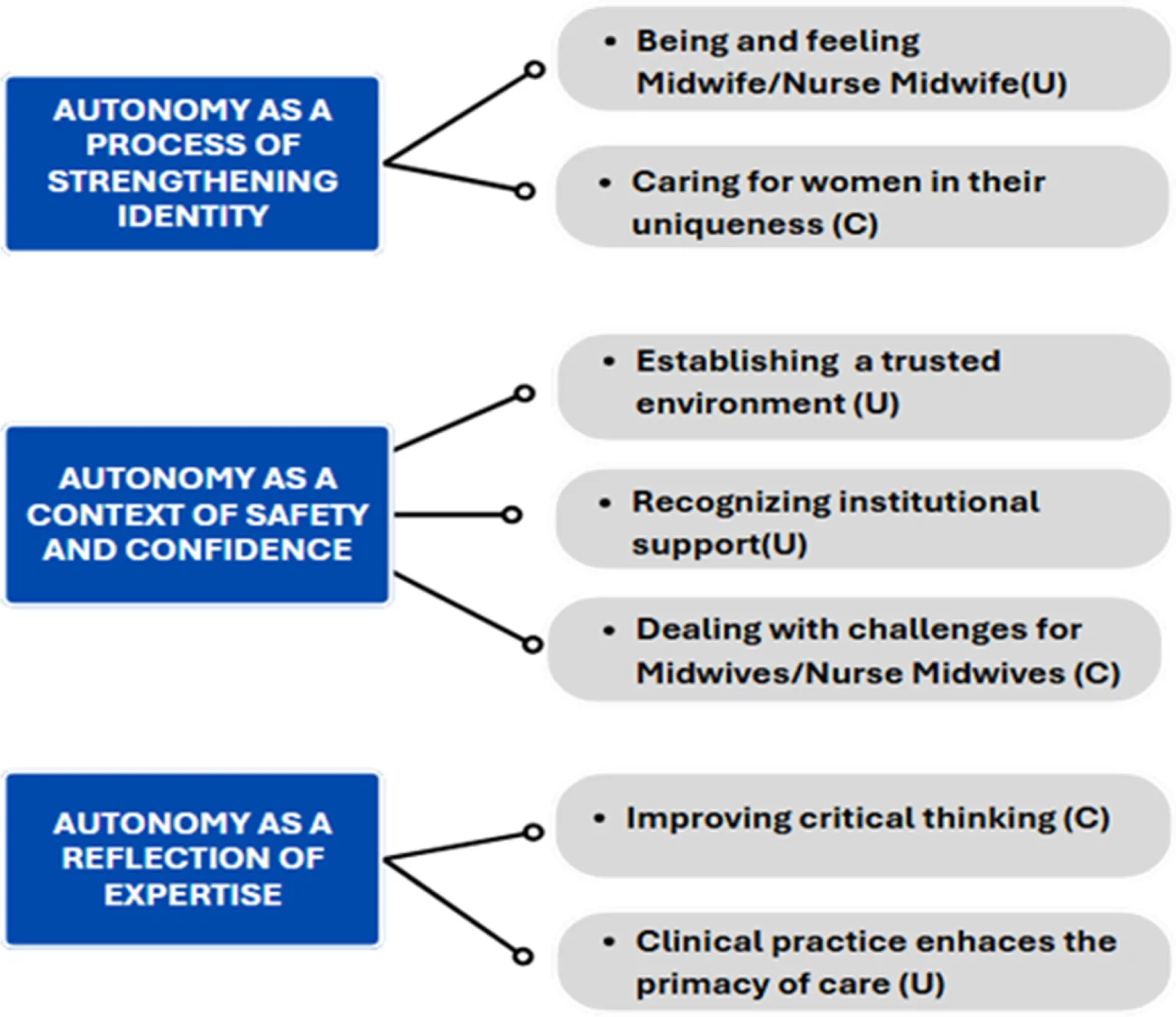
Meta-aggregation and metasynthesis results (U—unequivocal; C—credible).

The 67 findings extracted were aggregated into three synthesized categories: autonomy as a context of safety and confidence, autonomy as a process of strengthening identity, and autonomy as a reflection of expertise (Figure 2).

## Results

### Sample Characteristics

The metasynthesis included 16 articles published in nursing and/or obstetrics journals, conducted in eight countries: Australia (*n* = 3), the Netherlands (*n* = 3), Brazil (*n* = 2), Norway (*n* = 2), Iran (*n* = 2), South Africa (*n* = 1), Ireland (*n* = 1), Sweden (*n* = 1), and the United Kingdom (*n* = 1). A total of 216 M/NM working in health units were involved. Data collection was mainly conducted through in-depth interviews.

## Theme 1—Autonomy as a Context of Safety and Confidence

To express autonomy, it is necessary to preserve safety and confidence, and, according to the M/NM in the studies, this implies: establishing a trusted environment, recognizing institutional support, and dealing with challenges for M/NM.

### Establishing a Trusted Environment

A quality relationship based on understanding, knowledge, and trust promotes safety and positive birth experiences (Bradfield et al., 2019a; Remvik-Larsen et al., 2023). Through their holistic knowledge of women, autonomous M/NM provided effective and continuous care (Bradfield et al., 2019a). When faced with women in labor who expressed aspects of fear/satisfaction/confidence in the birth plan (Remvik-Larsen et al., 2023), autonomous, personalized, and respectful care management flowed (Bradfield et al., 2019a). However, for M/NM, giving birth autonomously required courage, overcoming difficulties, patience, and time (Ferreira Júnior et al., 2021). The brightness and sound of the delivery room, the shower, and the birthing balls were autonomous interventions. Thus, they created an atmosphere of safety and trust, which is essential for normal childbirth (Amiri-Farahani et al., 2022; Armanmehr et al., 2023). Women who had previously given birth in a hospital felt safer and more confident in units led by midwives. In this environment, M/NM could expand their autonomy, have a greater presence (Remvik-Larsen et al., 2023), and respond to women's needs (Bradfield et al., 2019a).

### Recognizing Institutional Support

Institutional support is essential for the autonomy of M/NM. The appreciation of institutional managers is a precursor to the empowerment, quality, and confidence of these professionals (Bradfield et al., 2019b; Oliveira et al., 2016). A disconcerting fact in delivery rooms was that M/NM from public institutions expressed greater autonomy in decisions than their colleagues in the private health system, but delivery was performed by obstetricians (Hastings-Tolsma et al., 2021), preventing M/NM from providing care due to financial interests. This made it impossible to practice the autonomous care model (Amiri-Farahani et al., 2022). Participants from public institutions reported that their autonomy was restricted by hospital management, contrary to national and/or international regulations, defending the technocratic model (Healy et al., 2017), hierarchical in medical protocols (Hastings-Tolsma et al., 2021). Institutions ignored physiological indicators, hid behind medical protocols, and skewed the interpretation of national/international standards. The concept of “normal” childbirth was destroyed, and the critical thinking of M/NM was extinguished (Ferguson et al., 2022). Only in units led by midwives did M/NM mention being fully autonomous in decision-making (Ferreira Júnior et al., 2021).

### Dealing with Challenges for M/NM

Several obstacles/challenges prevented M/NM from providing care autonomously. The lack of understanding/vision among women regarding childbirth as a psycho-physiological event was mentioned (Oliveira et al., 2016). This devaluation was exacerbated by financial issues, leading doctors to take over deliveries to avoid sharing revenue, thereby preventing M/NM from providing care. The independence and autonomy of M/NM were threatened by medical monitoring and control of low-risk deliveries. In hospitals, collaboration and trust among professionals turned into a power struggle (Aanensen et al., 2018). These differences/hierarchies between professionals (Armanmehr et al., 2023) led to difficulties in communication, collaboration, and trust in the M/NM–Woman–Obstetrician triangle (Amiri-Farahani et al., 2022). The participants’ increasing stress and workload had repercussions on task-based care models, which lacked holistic assistance, complying with medical prescriptions and thus leading to less autonomy.

## Theme 2—Autonomy as a Process of Strengthening Identity

Autonomy, as an identity-building process, is reinforced in the studies through an experience of autonomy lived daily, characterized by being and feeling a M/NM and caring for women in their uniqueness.

### Being and Feeling M/NM

Being and feeling like a midwife is identified as respecting the birth process, the life that is born, and the ability of women to give birth in their own time (Aanensen et al., 2018). M/NM described their profession as a unique art, as it is not reduced to technical procedures, but involves understanding women in the atmosphere of childbirth (Remvik-Larsen et al., 2023). An autonomous midwife would reassure and encourage women (Remvik-Larsen et al., 2023) and know how to be present (Bradfield et al., 2019a). M/NM who practiced in midwife-led units described tranquillity, but also presence, acting, when necessary, in contrast to the fast pace of hospitals, where anxiety about making mistakes, lack of support, and risk discourse prevented care (Spendlove, 2018). The profession and identity of M/NM were threatened by invasive and technological processes (Remvik-Larsen et al., 2023). An autonomous midwife would reassure and encourage women (Remvik-Larsen et al., 2023) and know how to be present (Bradfield et al., 2019a). M/NM who practiced in midwife-led units described tranquility, but also presence, acting, when necessary, in contrast to the fast pace of hospitals, where anxiety about making mistakes, lack of support, and risk discourse prevented care (Spendlove, 2018). The profession and identity of M/NM were threatened by invasive and technological processes (Remvik-Larsen et al., 2023). The biomedical model also incapacitated them, giving rise to defensive practice. Participants tailored care to women's needs (Remvik-Larsen et al., 2023). Their knowledge/experience, in line with the women's attitudes, influenced childbirth (Aanensen et al., 2018). Interventionist M/NM deprived women of being “owners” of their childbirth (Remvik-Larsen et al., 2023).

### Caring for Women in Their Uniqueness

Woman-centered care was safe, based on a relationship of trust and the individuality of the parturient (Amiri-Farahani et al., 2022; Bradfield et al., 2019a, 2019b). Prior knowledge of the woman's desired experience allowed for effective care during the intrapartum period, in a synchronous partnership (Bradfield et al., 2019a), quickly established (Amiri-Farahani et al., 2022), promoting normal childbirth (Remvik-Larsen et al., 2023). For M/NM, it was important to believe in the human nature of childbirth, especially in the perception of the laboring woman regarding her body and instinct/intuition (Remvik-Larsen et al., 2023). M/NM could enhance the skills and fulfillment of autonomous childbirth. The care of “being with the woman,” reinforced by the “continuity model,” gave M/NM greater security, through knowledge of the parturient, enabling them to anticipate problems, act, and change the care plan in response to needs (Bradfield et al., 2019a, 2019b). M/NM described two approaches to caring for the parturient, generating tension and creating controversy about the ideal practice (Nilsson et al., 2019). In the “being approach,” they focused on needs, prioritizing the wishes of laboring women, assuming an expectant attitude (L. Zondag et al., 2022), observing the details of labor and being at the woman's side with respect, trust, and autonomy (Remvik-Larsen et al., 2023). They were autonomous in managing labor, using technology and analgesia when justified (L. Zondag et al., 2022). In the “doing approach,” M/NM were busy with technical, pharmacological, or bureaucratic tasks, compromising care. In the “doing approach,” they focused on their duties, while in the “being approach,” they focused on the woman (Nilsson et al., 2019).

## Theme 3—Autonomy as a Reflection of Expertise

In the studies included, autonomy for M/NM constituted a field of knowledge that develops and progresses toward expertise through experience, namely, through improving critical thinking, and clinical practice enhances the primacy of care.

### Improving Critical Thinking

Assuming autonomy, M/NM felt confident in intervening if necessary/beneficial, in taking a wait-and-see approach to delivery (Nilsson et al., 2019), without routinely adhering to protocols (Ferguson et al., 2022). Participants who reported intervening frequently did not describe critical reflection in their practice (D. Zondag et al., 2022). Maintaining the quality of care and autonomy led M/NM to value safety through knowledge, case discussion, and common ideology, which is often challenged in hospital maternity wards (Remvik-Larsen et al., 2023). For the autonomy and decision-making of M/NM, it was also important to demonstrate communication skills with women and peers. Less interventionist M/NM reported greater openness in explaining procedures to women, understanding their questions and fears, fostering informed and shared decision-making, and providing personalized care (D. Zondag et al., 2022). In contrast, more interventionist M/NM limited information about interventions, skewing options, leading to a protocolized pattern (L. Zondag et al., 2022). In terms of interdisciplinary communication skills, the more autonomous participants exhibited greater capacity for argumentation and discussion, feeling more comfortable with disagreement from their peers (Healy et al., 2017). Their sole objective was to advocate for the women's wishes to promote a positive birth experience. In contrast, M/NM with less autonomy but more interventionist avoided confrontation with peers, especially obstetricians, yielding to their prescriptions and protocols (D. Zondag et al., 2022).

### Clinical Practice Enhances the Primacy of Care

Physiological childbirth is promoted by M/NM with knowledge, confidence, and experience (Remvik-Larsen et al., 2023), contrary to those who expect progression according to obstetric manuals and protocols (Aanensen et al., 2018). M/NM expressed frustration and abandonment of the profession due to the limitation of care autonomy imposed by a system that was inattentive to women's needs (Spendlove, 2018). Some M/NM were encouraged to ignore the individual experience of the parturient in favor of protocols/guidelines that justified the use of labor-accelerating maneuvers (Ferguson et al., 2022). Participants who submitted to the loss of autonomy, who did not show reluctance to their peers (Healy et al., 2017), feared consequences for themselves or for women (Weltens et al., 2019), experiencing tension between responsibility and accountability (Ferguson et al., 2022). Participants validated the final clinical fields of professional education/training as strongly influential in decision-making and interventionist attitudes (Weltens et al., 2019). By replicating learning, believing it to be the correct and acceptable care among peers, they conditioned their autonomy (D. Zondag et al., 2022). Less interventionist M/NM described greater reflection on experiences and greater use of evidence in decision-making. Confidence in midwifery grew with knowledge and experience (Armanmehr et al., 2023). Personalized care was decided autonomously in terms of interventions, detached from institutional protocols. Interventional participants denied using scientific evidence in their practice and decision-making (D. Zondag et al., 2022), relying instead on clinical protocols (Weltens et al., 2019).

## Discussion

This metasynthesis on understanding the experience of autonomy of M/NM in caring for parturients emphasized that autonomy as a context of safety and confidence, as a process of strengthening identity, and as a reflection of expertise are pillars that are supported by a dynamic experience for their self-improvement.

## Theme 1—Autonomy as a Context of Safety and Confidence

The findings highlighted the importance of a relationship of trust between the woman in labor and the M/NM. Autonomous M/NM promoted safety and confidence in laboring women and a perception of self-control during childbirth. They developed strategies to optimize instrumental techniques and human and empathetic relationships, focusing on the woman rather than the professionals. However, the autonomy of M/NM faces several obstacles already described (Sonmezer, 2021; Vermeulen, Buyl, et al., 2023; Zolkefli et al., 2020). The autonomy of M/NM suffered from financial interests and hierarchical subjugation in the public service, but with primacy in the private sector.

## Theme 2—Autonomy as a Process of Strengthening Identity

The studies included in the review described that the essence of being and feeling like a midwife goes beyond technical procedures, focusing on the art of respecting childbirth, understanding it as intrinsically human. The professional identity of M/NM is threatened by the biomedical model and by technical/technocratic processes, which lead to defensive practice and impact on a vision that is not based on evidence by society (Vermeulen, Fobelets, et al., 2023). M/NM with a weak sense of professional identity, due to the discrediting of their skills, were moving toward lower quality care and demotivation (Hunter et al., 2019). As a result, M/NM were discouraged from remaining in the profession (Zolkefli et al., 2020). In the findings, woman-centered care was considered the safest and most effective approach, which corroborates previous results (Clemons et al., 2021; Prosen, 2022; Vermeulen, Fobelets, et al., 2023).

## Theme 3—Autonomy as a Reflection of Expertise

In the evidence, critical thinking and practical experience were vital for M/NM's autonomy, allowing them to question interventions and protocols (Zolkefli et al., 2020). Less interventionist M/NM demonstrated this ability through reflection, discussion, or knowledge sharing, expressing confidence and assurance. Communication was another skill that facilitated decision-making. Autonomous M/NM were consistent between clarifying procedures and the resulting informed decision of the parturient (Zolkefli et al., 2020). In contrast, interventionist M/NM, but fearful of reprisals, avoided confrontation and yielded to protocols and medical prescriptions. There is an urgent need for uniform international health policies oriented toward less interventionist models (i.e., midwife-led care) and protocols that advocate for the physiology of childbirth rather than the “supposed” risk, a biased justification for manipulating the process (Clemons et al., 2021). In midwifery education, high-quality curricula and faculty support are significant indicators (Aqtam et al., 2025) for confident and autonomous professional practice. Clinical teaching in M/NM-led units are crucial experience, promoting reflective practice, critical thinking, and decision-making. Students can thus understand the concept of autonomy and learn the role of M/NM in a changing culture (Vermeulen et al., 2024).

**Figure 3.**
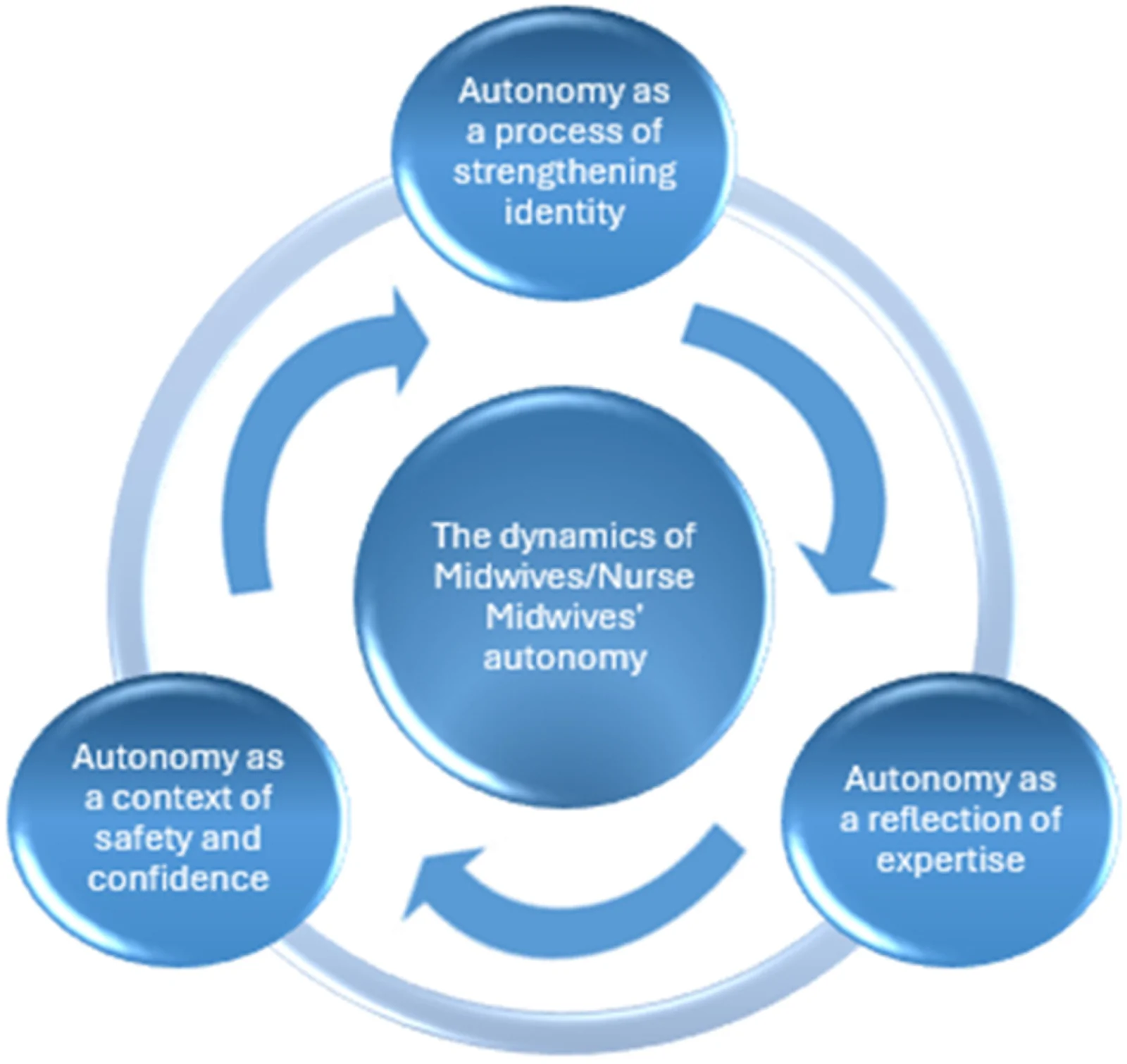
List of themes identified.

The three themes are continuously and interdependently linked, illustrating that the autonomy of M/NM is a conceptualization of professional practice that is dynamic and multifaceted, shaped, nourished, and fed back by identity, expertise, and the context of practice (Figure 3).

This dynamic stems from M/NM, who, by exercising their autonomy, contribute to building and strengthening their professional identity by taking ownership of their role through independent and competent decision-making. They earn their place in multidisciplinary teams, institutions, and social recognition.

Expertise highlights the technical pillar that underpins the dynamics of M/NM autonomy. Whether through continuing education or practical experience, the expertise of M/NM reflects the knowledge and competence that gives them authority in decision-making and underpins autonomous practice. The latter is only possible in an environment of safety, trust, and respect between peers and parturients, which allows for responsible practice. The context of practice, by allowing the autonomy of M/NM, strengthens their role and professional identity and improves their expertise, restarting the process. When the context is adverse, the cycle becomes negative, weakening identity and limiting development.

In short, the autonomy of M/NM is a dynamic, cyclical, continuous process of professional growth and consolidation in all dimensions of practice. Autonomy, when realized in decision-making and Socratic philosophical eloquence, is the “arete” (virtue) of acting well (Ferrari, 2014).

## Strengths and Limitations

- This is the first systematic review with a metasynthesis on the experiences of autonomy of M/NM in caring for laboring women in health facilities.
- The metasynthesis complies with the guidelines recommended for its preparation, and its conclusions strengthen the autonomous practice of M/NM.
- The limitation of this review is the geographical scope of the studies included, which may limit the transferability of the results.

## Implications for Practice

To respond effectively to maternal health issues, it is imperative to develop inter- or transdisciplinary training models that promote mutual knowledge of skills among professionals and fully autonomous practice.

Development of M/NM-led units in hospitals, as they reinforce the autonomy of these professionals and improve the quality, safety, and satisfaction of laboring women. This type of care model will challenge established hierarchies and move towards collaborative contexts that value M/NM.

In the discipline of Nursing, the findings reaffirm its own body of knowledge, decision-making based on scientific evidence, and the continuity of person-centered care practice. This metasynthesis proves that for Maternal Health Nursing, professional autonomy is a vector that requires continuous investment in advanced training, the development of complex skills, and the robustness of professional identity.

The scientific community should continue the discussion in this area of knowledge to strengthen emerging changes in health policies. Autonomous care by M/NM is the path to sustainability, quality, and safety in global maternity care.

Recognizing the autonomy of M/NM multiplies health gains. In addition to professional, individual, and collective benefits, it enhances health as an invaluable asset.

## Conclusion

The experience of autonomy of M/NM in caring for laboring women is linked to three dynamically related issues: the context of care, identity, and expertise. By “being and doing” as an expression of their autonomy, M/NM demonstrate their knowledge/skills and experience and legitimize a model of care based on trust, safety, and the physiology of childbirth, transforming the birth setting into a space of protection and respect. Investment in the autonomy of M/NM at the institutional, political, and social levels is essential.

## Supplemental Material

sj-docx-1-son-10.1177_23779608261433120 - Supplemental material for Midwives’ and Nurse Midwives’ Autonomy Experience in Caring for Laboring Women in Health Facilities: A Systematic Review With Metasynthesis Supplemental material, sj-docx-1-son-10.1177_23779608261433120 for Midwives’ and Nurse Midwives’ Autonomy Experience in Caring for Laboring Women in Health Facilities: A Systematic Review With Metasynthesis by Elsa Salgueiro, Catarina Afonso, Margarida Sim-Sim, Claúdia Janeiro and Maria Otília Zangão in SAGE Open Nursing

sj-docx-2-son-10.1177_23779608261433120 - Supplemental material for Midwives’ and Nurse Midwives’ Autonomy Experience in Caring for Laboring Women in Health Facilities: A Systematic Review With Metasynthesis Supplemental material, sj-docx-2-son-10.1177_23779608261433120 for Midwives’ and Nurse Midwives’ Autonomy Experience in Caring for Laboring Women in Health Facilities: A Systematic Review With Metasynthesis by Elsa Salgueiro, Catarina Afonso, Margarida Sim-Sim, Claúdia Janeiro and Maria Otília Zangão in SAGE Open Nursing

sj-docx-3-son-10.1177_23779608261433120 - Supplemental material for Midwives’ and Nurse Midwives’ Autonomy Experience in Caring for Laboring Women in Health Facilities: A Systematic Review With Metasynthesis Supplemental material, sj-docx-3-son-10.1177_23779608261433120 for Midwives’ and Nurse Midwives’ Autonomy Experience in Caring for Laboring Women in Health Facilities: A Systematic Review With Metasynthesis by Elsa Salgueiro, Catarina Afonso, Margarida Sim-Sim, Claúdia Janeiro and Maria Otília Zangão in SAGE Open Nursing

sj-docx-4-son-10.1177_23779608261433120 - Supplemental material for Midwives’ and Nurse Midwives’ Autonomy Experience in Caring for Laboring Women in Health Facilities: A Systematic Review With Metasynthesis Supplemental material, sj-docx-4-son-10.1177_23779608261433120 for Midwives’ and Nurse Midwives’ Autonomy Experience in Caring for Laboring Women in Health Facilities: A Systematic Review With Metasynthesis by Elsa Salgueiro, Catarina Afonso, Margarida Sim-Sim, Claúdia Janeiro and Maria Otília Zangão in SAGE Open Nursing

sj-docx-5-son-10.1177_23779608261433120 - Supplemental material for Midwives’ and Nurse Midwives’ Autonomy Experience in Caring for Laboring Women in Health Facilities: A Systematic Review With Metasynthesis Supplemental material, sj-docx-5-son-10.1177_23779608261433120 for Midwives’ and Nurse Midwives’ Autonomy Experience in Caring for Laboring Women in Health Facilities: A Systematic Review With Metasynthesis by Elsa Salgueiro, Catarina Afonso, Margarida Sim-Sim, Claúdia Janeiro and Maria Otília Zangão in SAGE Open Nursing

## References

[bibr1-23779608261433120] Aanensen E. H. Skjoldal K. Sommerseth E. Dahl B. (2018). Easy to believe in, but difficult to carry out—Norwegian Midwives’ experiences of promoting normal birth in an obstetric-led maternity unit. International Journal of Childbirth, 8(3), 167–176. 10.1891/2156-5287.8.3.167

[bibr2-23779608261433120] Amiri-Farahani L. Gharacheh M. Sadeghzadeh N. Peyravi H. (2022). Iranian Midwives’ lived experiences of providing continuous midwife-led intrapartum care: A qualitative study. BMC PREGNANCY AND CHILDBIRTH, 22(22:724), 1–10. 10.1186/s12884-022-05040-z 36151533 PMC9502875

[bibr3-23779608261433120] Aqtam I. Shouli M. Hmod H. Torman S. Salman R. (2025). Satisfaction and self-confidence levels among midwifery students in clinical training programmes at multisite Palestinian universities: A cross-sectional study. BMJ Open, 15(9), e096845. 10.1136/bmjopen-2024-096845 PMC12481396 40998443

[bibr4-23779608261433120] Armanmehr V. Banafshe E. Hashemizadeh H. Alami A. Khajavi A. (2023). Midwives’ perception regarding conditions influencing the implementation of the natural childbirth promotion program in Iran: A qualitative study. International Journal of Community Based Nursing and Midwifery, 11(2), 135–148. mdc. 10.30476/IJCBNM.2023.97165.2169 37114101 PMC10126447

[bibr5-23779608261433120] Bradfield Z. Kelly M. Hauck Y. Duggan R. (2019a). it’s what midwifery is all about’: Western Australian midwives’ experiences of being “with woman” during labour and birth in the known midwife model. BMC Pregnancy and Childbirth, 19(1), 29. 10.1186/s12884-018-2144-z 30642287 PMC6332887

[bibr6-23779608261433120] Bradfield Z. Kelly M. Hauck Y. Duggan R. (2019b). Midwives’ perceptions of being ‘with woman’: A phenomenological study. BMC Pregnancy and Childbirth, 19(1), 363. 10.1186/s12884-019-2548-4 31638923 PMC6802311

[bibr7-23779608261433120] Clemons J. H. Gilkison A. Mharapara T. L. Dixon L. McAra-Couper J. (2021). Midwifery job autonomy in New Zealand: I do it all the time. Women and Birth: Journal of the Australian College of Midwives, 34(1), 30–37. 10.1016/j.wombi.2020.09.004 32962945

[bibr8-23779608261433120] Faria P. Camargo D. (2022). Metassíntese: Revisão sistemática qualitativa na área da educação. Revista Brasileira de Educação, 27(1), e270122. 10.1590/S1413-24782022270122

[bibr9-23779608261433120] Ferguson B. Baldwin A. Henderson A. Harvey C. (2022). The grounded theory of coalescence of perceptions, practice and power: An understanding of governance in midwifery practice. Journal of Nursing Management, 30(8), 4587–4594. Scopus. 10.1111/jonm.13892 36325759 PMC10099921

[bibr10-23779608261433120] Ferrari F. (2014). Conhecimento e virtude no mênon de platão. Archai, 12(Jan-Jun), 81–87. 10.14195/1984-249X_12_8

[bibr11-23779608261433120] Ferreira Júnior A. R. dos Santos Brandão L. C. Carolina de Melo Farias Teixeira A. Ramos Cardoso A. M. (2021). Potentialities and limitations of nurses’ performance in the normal birth center. Anna Nery School Journal of Nursing / Escola Anna Nery Revista de Enfermagem, 25(2), 1–8. 10.1590/2177-9465-EAN-2020-0080

[bibr12-23779608261433120] Hastings-Tolsma M. Temane A. Tagutanazvo O. B. Lukhele S. Nolte A. G. (2021). Experience of midwives in providing care to labouring women in varied healthcare settings: A qualitative study. Health SA Gesondheid, 26(1), a1524. 10.4102/hsag.v26i0.1524 PMC8182560 34192066

[bibr13-23779608261433120] Healy S. Humphreys E. Kennedy C. (2017). A qualitative exploration of how midwives’ and obstetricians’ perception of risk affects care practices for low-risk women and normal birth. Women & Birth, 30(5), 367–375. CINAHL Plus with Full Text. 10.1016/j.wombi.2017.02.005 28279637

[bibr14-23779608261433120] Hunter B. Fenwick J. Sidebotham M. Henley J. (2019). Midwives in the United Kingdom: Levels of burnout, depression, anxiety and stress and associated predictors. Midwifery, 79(December), 102526. 10.1016/j.midw.2019.08.008 31473405

[bibr15-23779608261433120] Lockwood C. Munn Z. Porritt K. (2015). Qualitative research synthesis: Methodological guidance for systematic reviewers utilizing meta-aggregation. JBI Evidence Implementation, 13(3), 179. 10.1097/XEB.0000000000000062 26262565

[bibr16-23779608261433120] Moher D. Shamseer L. Clarke M. Ghersi D. Liberati A. Petticrew M. Shekelle P. Stewart L. A. , & PRISMA-P Group. (2015). Preferred reporting items for systematic review and meta-analysis protocols (PRISMA-P) 2015 statement. Systematic Reviews, 4(1), 1. 10.1186/2046-4053-4-1 25554246 PMC4320440

[bibr17-23779608261433120] Munn Z. Porritt K. Lockwood C. Aromataris E. Pearson A. (2014). Establishing confidence in the output of qualitative research synthesis: The ConQual approach. BMC Medical Research Methodology, 14(1), 108. 10.1186/1471-2288-14-108 25927294 PMC4190351

[bibr18-23779608261433120] Nilsson C. Olafsdottir O. A. Lundgren I. Berg M. Dellenborg L. (2019). Midwives’ care on a labour ward prior to the introduction of a midwifery model of care: A field of tension. International Journal of Qualitative Studies on Health and Well-Being, 14(1), 1593037. 10.1080/17482631.2019.1593037 30924414 PMC6450488

[bibr19-23779608261433120] Oliveira J. Campo T. Souza F. Davim R. Dantas J. (2016). Obstetric nurses’perception in assistance to the parturient. Journal of Nursing UFPE / Revista de Enfermagem UFPE, 10(10), 3868–3875. 10.5205/reuol.9667-87805-1-ED1010201619

[bibr20-23779608261433120] Ouzzani M. Hammady H. Fedorowicz Z. Elmagarmid A. (2016). Rayyan – A web and mobile app for systematic reviews. Systematic Reviews, 5(1), 210. 10.1186/s13643-016-0384-4 27919275 PMC5139140

[bibr21-23779608261433120] Page M. J. McKenzie J. E. Bossuyt P. M. Boutron I. Hoffmann T. C. Mulrow C. D. Shamseer L. Tetzlaff J. M. Akl E. A. Brennan S. E. Chou R. Glanville J. Grimshaw J. M. Hróbjartsson A. Lalu M. M. Li T. Loder E. W. Mayo-Wilson E. McDonald S , …, D. Moher (2021). The PRISMA 2020 statement: An updated guideline for reporting systematic reviews. BMJ (Clinical Research Ed.), 372(March), 71. 10.1136/bmj.n71 PMC8005924 33782057

[bibr22-23779608261433120] Prosen M. (2022). A systematic integrative literature review of the factors influencing the professionalization of midwifery in the last decade (2009–2019). Midwifery, 106(March), 103246. 10.1016/j.midw.2021.103246 35007977

[bibr23-23779608261433120] Remvik-Larsen L. Gran A. M. W. Dahl B. (2023). Midwives’ experiences of facilitating normal birth in midwifery-led units in Norway: A qualitative study. European Journal of Midwifery, 7(December), 1–8. 10.18332/ejm/173388 38090368 PMC10711833

[bibr24-23779608261433120] Sandelowski M. (2012). Metasynthesis of qualitative research. In APA Handbook of research methods in psychology, vol 2: Research designs: Quantitative, qualitative, neuropsychological, and biological (pp. 19–36). American Psychological Association. 10.1037/13620-002

[bibr25-23779608261433120] Sonmezer E. (2021). Professional autonomy for midwives in the contemporary UK maternity system: Part 2. British Journal of Midwifery, 29(1), 19–25. 10.12968/bjom.2021.29.1.19

[bibr26-23779608261433120] Spendlove Z. (2018). Risk and boundary work in contemporary maternity care: Tensions and consequences. Health, Risk &amp; Society, 20(1/2), 63–80. https://www.britannica.com/topic/autonomy. https://doi.org/10.1080/13698575.2017.1398820

[bibr27-23779608261433120] UNFPA, ICM, WHO. (2021). *The State of the World’s Midwifery* 2021. https://www.who.int/publications/i/item/sowmy_2021.

[bibr28-23779608261433120] Vermeulen J. Buyl R. Luyben A. Fleming V. Fobelets M. (2022). Defining midwifery autonomy in Belgium: Consensus of a modified delphi study. JOURNAL OF ADVANCED NURSING, 78(9), 2849–2860. 10.1111/jan.15209 35266191

[bibr29-23779608261433120] Vermeulen J. Buyl R. Luyben A. Fleming V. Fobelets M. (2023). Key maternity care Stakeholders’ views on Midwives’ professional autonomy. Healthcare (Basel, Switzerland), 11(9), 1231. 10.3390/healthcare11091231 37174773 PMC10177750

[bibr30-23779608261433120] Vermeulen J. Buyl R. Luyben A. Fleming V. Tency I. Fobelets M. (2024). The exploration of professional midwifery autonomy: Understanding and experiences of final-year midwifery students. Nurse Education Today, 134(March), 106101. 10.1016/j.nedt.2024.106101 38266429

[bibr31-23779608261433120] Vermeulen J. Fobelets M. Fleming V. Luyben A. Stas L. Buyl R. (2023). How do midwives view their professional autonomy, now and in future? Healthcare, 11(12), 1800. 10.3390/healthcare11121800 37372919 PMC10297976

[bibr32-23779608261433120] Webler N. de Almeida L. C. G. Carneiro J. B. Campos L. M. Glaeser T. A. Couto T. M. de Lima S. G. Silva I. B. (2023). Professional autonomy in dealing with complications: Discourse of obstetric nurses working in planned home births. Revista Brasileira De Enfermagem, 76(2), e20220388. 10.1590/0034-7167-2022-0388 PMC10021941 36946813

[bibr33-23779608261433120] Weltens M. de Nooijer J. Nieuwenhuijze M. J. (2019). Influencing factors in midwives’ decision-making during childbirth: A qualitative study in The Netherlands. Women and Birth, 32(2), e197–e203. Scopus. 10.1016/j.wombi.2018.06.009 29935989

[bibr34-23779608261433120] WHO, ICM, WRA. (2016). *Midwives’ Voices Midwives’ Realities*. https://www.who.int/publications/i/item/9789241516112.

[bibr35-23779608261433120] Zolkefli Z. H. H. Mumin K. H. A. Idris D. R. (2020). Autonomy and its impact on midwifery practice. British Journal of Midwifery, 28(2), 120–129. 10.12968/bjom.2020.28.2.120

[bibr36-23779608261433120] Zondag D. van Haaren-Ten Haken T. Offerhaus P. Maas V. Nieuwenhuijze M. (2022). Knowledge and skills used for clinical decision-making on childbirth interventions: A qualitative study among midwives in The Netherlands. European Journal of Midwifery, 6(September), 1–9. 10.18332/ejm/151653 PMC9434498 36119405

[bibr37-23779608261433120] Zondag L. Maas V. Beuckens A. Nieuwenhuijze M. (2022). Experiences, beliefs, and values influencing Midwives’ attitudes toward the use of childbirth interventions. Journal of Midwifery and Women’s Health, 67(5), 618–625. 10.1111/jmwh.13392 PMC9805008 35916393

