# Supplementary material for: Midwives’ and Nurse Midwives’ Autonomy Experience in Caring for Laboring Women in Health Facilities: A Systematic Review With Metasynthesis
Source: SAGE Open Nurs. 2026 Sep 7;12:23779608261433120. doi: 10.1177/23779608261433120 (PMC13554601; doi:10.1177/23779608261433120)
Supplement: Supplementary file 1 [file sj-docx-1-son-10.1177_23779608261433120.docx]

**Appendix I. Critical appraisal results of eligible studies**

| **Study** | **Q.1** | **Q.2** | **Q.3** | **Q.4** | **Q.5** | **Q.6** | **Q.7** | **Q.8** | **Q.9** | | **Q.10** | | **QARI** | |
| --- | --- | --- | --- | --- | --- | --- | --- | --- | --- | --- | --- | --- | --- | --- |
| Remvik-Larsen, L **(2023)** | Y | Y | Y | Y | Y | N | U | Y | Y | Y | | **80%** | |  |
| Armanmehr V **(2023)** | U | Y | Y | U | Y | Y | Y | Y | Y | Y | | **80%** | |  |
| Amiri-Farahani, L **(2022)** | Y | Y | Y | Y | Y | Y | Y | U | Y | Y | | **90%** | |  |
| Ferguson, B **(2022)** | Y | Y | Y | Y | Y | Y | Y | U | Y | Y | | **90%** | |  |
| Zondag D **(2022)** | Y | Y | Y | Y | Y | U | Y | U | Y | Y | | **80%** | |  |
| Zondag, L **(2022)** | Y | Y | Y | Y | Y | U | Y | U | Y | Y | | **80%** | |  |
| Hastings-Tolsma M **(2021)** | Y | Y | Y | Y | Y | Y | Y | Y | Y | Y | | **100%** | |  |
| Ferreira Júnior, A **(2021)** | Y | Y | Y | Y | Y | U | U | Y | Y | Y | | **80%** | |  |
| Bradfield Z **(2019)a** | Y | Y | Y | Y | Y | Y | Y | Y | Y | Y | | **100%** | |  |
| Bradfield Z **(2019)b** | Y | U | Y | Y | Y | Y | Y | Y | Y | Y | | **90%** | |  |
| Weltens, M **(2019)** | Y | Y | Y | Y | Y | N | Y | Y | Y | Y | | **90%** | |  |
| Nilsson, C **(2019)** | Y | Y | Y | Y | Y | Y | Y | N | Y | U | | **80%** | |  |
| Spendlove, Z **(2018)** | Y | U | Y | Y | Y | Y | Y | Y | Y | Y | | **90%** | |  |
| Aanensen, E **(2018)** | Y | Y | Y | Y | Y | Y | U | Y | Y | Y | | **90%** | |  |
| Healy, S **(2017)** | Y | Y | Y | Y | Y | Y | Y | Y | U | Y | | **90%** | |  |
| Oliveira, J **(2016)** | Y | Y | Y | Y | Y | U | U | Y | Y | Y | | **80%** | |  |
|  | 90% | 80% | 100% | 90% | 100% | 40% | 60% | 50% | 90% | 90% | |  | |  |
| Y-yes; N-no; U-unclear |  |  |  |  |  |  |  |  |  |  | |  | |  |
