# Supplementary material for: Midwives’ and Nurse Midwives’ Autonomy Experience in Caring for Laboring Women in Health Facilities: A Systematic Review With Metasynthesis
Source: SAGE Open Nurs. 2026 Sep 7;12:23779608261433120. doi: 10.1177/23779608261433120 (PMC13554601; doi:10.1177/23779608261433120)
Supplement: Supplementary file 2 [file sj-docx-2-son-10.1177_23779608261433120.docx]

| **Author Year**  **Appendix I. Description of Included Articles** | **Country** | **Study title** | **Objective** | **Methodology**  **Methods** | **Population**  **Setting**  **Sample** | **Results** |
| --- | --- | --- | --- | --- | --- | --- |
| Remvik-Larsen et al. (2023) | Norway | Midwives' experiences of facilitating normal birth in midwifery-led units in Norway: A qualitative study | To gain a deeper understanding of midwives’ experiences of facilitating normal birth in midwifery-led units | - Phenomenology - Semi-structured interviews | - 7 midwives - Midwifery-led unit - Purposive in Facebook | - The importance of relationships, continuity, and a safe atmosphere - Midwife in the hand, in the heart, and in the mind - Confidence in the physiological process of childbirth, midwifery autonomy, and a common ideology |
| Armanmehr et al. (2023) | Iran | Midwives’ perception regarding conditions influencing the implementation of the Natural  Childbirth Promotion Program in Iran: A Qualitative Study | To explore the perceptions of midwives about conditions influencing the implementation of Natural Childbirth Promotion Program (NCPP) | - Descriptive and exploratory study - Depth interviews - Thematic analysis | - 12 midwives - Public units - Purposive | - Responsive staff - Characteristics of the parturient - Recognition of midwifery role - Teamwork - The birthing environment - Effective management - Institutional and social contexto - Social education |
| Amiri-Farahani et al. (2022) | Iran | Iranian midwives’ lived experiences of providing continuous midwife‑led intrapartum care: a qualitative study | To explore midwives' lived experiences of delivering continuous midwife-led intrapartum care | - Phenomenology - Descriptive phenomenology | - 10 midwives - Public and private units | - Emphasis on the non‑interventional care - Midwifery-specific focus - Obstacles and challenges of midwifery care - Perceptions and behaviours - Shifting practice |
| Ferguson et al. (2022) | Australia | The grounded theory of Coalescence of Perceptions, Practice and Power: An understanding of governance in midwifery | To understand midwifery care during labour, particularly decision-making processes, within Australian health systems. | - Grounded theory - Semi-structured interviews - Straussian grounded theory | - 18 midwives - Hospital and midwifery-led unit | - Compliance - Power within practice |
| D. Zondag et al. (2022) | Netherlands | Knowledge and skills used for clinical decision-making on childbirth interventions: A qualitative study among midwives in the Netherlands | To explore how knowledge and skills influence clinical decision-making of midwives on the appropriate use of childbirth interventions. | - Qualitative - In-depth interviews that combined a narrative approach with a semi-structured question route - Deductive content analysis | - 20 midwives - Primary care - Purposive | - Knowledge - learning from different sources - Critical thinking skills – advanced knowledge in context - Communication skills – making knowledge work |
| L. Zondag et al. (2022) | Netherlands | Experiences, beliefs, and values influencing midwives’  attitudes toward the use of childbirth interventions | To explore experiences, beliefs and values that influence midwives' attitudes toward intervention in perinatal care | - Qualitative - Semi-structured interviews - Inductive content analysis | - 20 midwives - Primary care | - Attitudes toward interventions - Influences on midwives’ attitudes - Supportive and directive styles |
| Hastings-Tolsma et al. (2021) | South Africa | Experience of midwives in providing care to labouring women in varied healthcare settings: A qualitative study | To explore and describe the experiences of midwives in providing care to labouring women in varied healthcare settings | - Qualitative - Semi-structured interviews in-depth | - 10 midwives - Purposive, convenience | - Proud to be a midwife - Regulations and independent function - Resource availability - Work burden - Image of the midwife |
| Ferreira Júnior et al. (2021) | Brazil | Potentialities and limitations of nurses’ performance in the Normal Birth Center | To know the potentialities and limitations of the nurse’s performance in the Center for Normal Birth (CPN) | - Descriptive and exploratory study - Depth interviews - Thematic analysis - Sociology of professions | - 6 nurse midwives - Normal childbirth center intra-hospital | - Knowledge and autonomy - Credentialism - Work division - Table of values |
| Bradfield et al. (2019)a | Australia | It's what midwifery is all about": Western Australian midwives' experiences of being 'with woman' during labour and birth in t | To explore the experiences of being ‘with woman’ during labour and birth from the perspective of midwives’ working in a model where care is provided by a known midwife. | - Phenomenology - Depth interviews - Descriptive phenomenology | - 10 midwives - Public units - Purposive, convenience snowball | - Building relationships - Woman centred care that is safe - Impact on the midwife - Impact on the Woman - Challenges in the ‘known midwife’ model along with corresponding subthemes |
| Bradfield et al. (2019)b | Australia | Midwives’ perceptions of being ‘with woman’: A phenomenological study. | To describe Western Australian midwives' perceptions of the phenomenon of being 'with woman' during the intrapartum period | - Phenomenology - Depth interviews - Descriptive phenomenology (Giorgi) | - 31 midwives - Public and private - Purposive, snowball | - Essential to professional identity - Partnership - Woman-centred practice |
| Weltens et al. (2018) | Netherlands | Influencing factors in midwives' decision-making during childbirth: A qualitative study in the Netherlands | To gain understanding of underlying factors in the decision-making process to referral to obstetric-led care among midwives attending childbirth in midwifery-led care | - Phenomenology - Depth interviews - Thematic analysis | - 10 midwives - Midwifery-led unit - Purposive | - Knowledge - Experience - Environment - Influence of others - Subjective factors |
| Nilsson et al. (2019) | Sweden | Midwives’ care on a labour ward prior to the introduction of a midwifery model of care: a field of tension | To explores the content and meaning of midwives’ care of women in a hospital-based labour ward in Sweden prior to the introduction of a theoretical midwifery model of care | - Ethnography - Participant observation and 2 in-depth interviews | - 6 midwives - Hospital | - The birthing rooms and the office: different rooms of care - Women giving birth or being delivered: midwives’ expectations and relationships with women - Old and new caring roles of the midwife: women giving birth in a “new age” - Being and doing: different approaches to caring - Holistic and reductionist care: guided by contrasting models and guidelines |
| Spendlove (2018) | United Kingdom | Risk and boundary work in contemporary maternity care: tensions and consequences | To explore the risks of tensions at work, in the context of contemporary maternity care, in relation to the limits of the professional roles of obstetricians and midwives | - Ethnography - Participant observation and semi -structured interviews in-depth - Thematic analysis | - 21 midwives - 16 obstetricians - Obstetric-led maternity - Purposive | - Anxiety over uncertainty, error and subsequent blame provoking defensive practice - Tension of risk work: risk minimisation versus risk causation - Shifting of professional role boundaries |
| Aanensen et al. (2018) | Norway | Easy to believe in, but difficult to carry out — Norwegian  midwives’ experiences of Promoting Normal Birth in an obstetric-led maternity unit | To explore and describe midwives' experiences of promoting normal birth in medicalized obstetric-led births units | - Descriptive and exploratory study - Semi-structured interviews - Systematic text | - 10midwives - 2 obstetric-led birth units - Convenience | - How Midwives’ perceptions of their own as well as the women’s personal attributes and attitudes influence the birthing process - Lack of time and impatient staff negatively affect the woman’s chances of giving birth normally - Increasing use of procedures, selection criteria, and technology threatens midwifery skills and prevents midwives from promoting normal births |
| Healy et al. (2017) | Ireland | A qualitative exploration of how midwives’ and obstetricians’ perception of risk affects care practices for low-risk women and normal birth | To understand midwives’ and obstetricians’ perceptions of risk regarding low-intervention birth; To investigate how this affects decision-making | - Grounded theory - Semi-structured interviews in-depth - Thematic analysis | - 16 midwives - 9 obstetricians - Purposive | - Professional autonomy and hierarchy in maternity care - Midwifery-led care as an undervalued and unsupported aspiration - A shift in focus from striving for normality to risk management - Viewing pregnancy through a risk-lens |
| Oliveira et al. (2016) | Brazil | Obstetric nurses´ perception in assistance to the parturient | To understand obstetric nurses' perceptions of their role in caring for parturient in a public maternity hospital | - Descriptive study - Semi-structured interviews - Thematic analysis | - 9 nurse midwives | - Assistance activities - Health education - Institutional management support - Multidisciplinary teamwork |
