# Supplementary material for: Midwives’ and Nurse Midwives’ Autonomy Experience in Caring for Laboring Women in Health Facilities: A Systematic Review With Metasynthesis
Source: SAGE Open Nurs. 2026 Sep 7;12:23779608261433120. doi: 10.1177/23779608261433120 (PMC13554601; doi:10.1177/23779608261433120)
Supplement: Supplementary file 3 [file sj-docx-3-son-10.1177_23779608261433120.docx]

Appendix III. Data extraction

|  | | **Review question:**  What are the lived experiences (I) of Midwives/Nurse Midwives (P) regarding autonomy in caring for laboring women in health facilities (C)? | | | | |
| --- | --- | --- | --- | --- | --- | --- |
|  |  | **Findings** | **Illustrations** | **Evidence*** | | |
|  |  |  |  | **Unequivocal** | **Credible** | **Unsupported** |
| 1. Remvik-Larsen L, Gran A, Dahl B (2023) | 1 | The importance of relationships, continuity, and a safe atmosphere | “The first thing I think, which I always have in the back of my mind, is what can I as a midwife do to make this woman feel safe and well cared for? How can I ensure that she can be herself when she is giving birth to her child, and that I can give her, to the greatest extent possible, what she needs and wants? I have to “tune in” to this woman, to the woman in labor.” (Hide) | U |  |  |
|  | 2 | Midwife in the hand, in the heart, and in the mind | “I hear how people breathe, and I hear when their contractions start and stop, and I see how the women move, so that's my most important instrument. I don't have to listen so much to fetal sounds when I know she has good breaks between contractions. Because that tells me that the baby is getting oxygen in between times.” (Grethe) | U |  |  |
|  | 3 | Confidence in the physiological process of childbirth, midwifery autonomy, and a common ideology | “You tend to cheat a bit on the partograph, yeah. And you have to do that because otherwise hardly anyone would be able to give birth in peace. And don't open the partograph too early. You can of course be lucky if you do that, but it's a bit sad that it has to be like that.” (Nina) | U |  |  |
| 2. Zondag D, van Haaren-Ten Haken T, Offerhaus P, Maas V, Nieuwenhuijze M (2022) | 4 | Knowledge - learning from different sources   a) *Getting the basics* b) *Deepening the knowledge base*  - *Learning through experience* - *Reflection on evidence* | “They [educators] really taught me to act according to the physiology. They also taught me to show what you can do as a primary care midwife.” (Midwife 7)  “The longer you work, the easier it becomes to feel free in decision-making and to decide what is best in each situation” (Midwife 12)  “In our practice we say “the less you do, the more beautiful the birth will be”. It's a kind of conscious choice […] It's also very clearly written in the Lancet series. A lot of midwives use amniotomy to accelerate labor, and then you think: “why would you want to do that?” […] Eventually, it becomes evident that it does not help at all.” (Midwife 1) | U |  |  |
|  | 5 | Critical thinking skills – advanced knowledge in  context | “During childbirth I try to say to myself: “hold back, you don't have to [artificial rupture the membranes]. The woman does not benefit from that. She will only have more severe contractions. That's of no benefit”.” (Midwife 20) | U |  |  |
|  | 6 | Communication skills – making knowledge work   - *Communication with women: giving and gaining background information*  a) *Instrumental communication* b) *Affective communication*  - *Communication with colleagues: the ability to speak up* | “In general, we find it easier to give more [interventions] than to give less [interventions]. Healthcare providers see more danger in not intervening than in intervening. I think it is my job to make this clear to the woman and her partner.” (Midwife 12)  “Someone may say that she wants to give birth at home, even when it's preterm. But then I still know nothing. Is she traumatized? Would she like to experience this once? […] Actually, the conversation starts at that point. You need to know a lot more.”  (Midwife 12)  “During multidisciplinary meetings we are transparent and open. We honestly say what we think and what we want. […] Always as the advocate of the woman. It is very important that you neatly organize the desired healthcare plan for her.” (Midwife 1) | U |  |  |
| 3. Hastings-Tolsma M, Temane A, Tagutanazvo O, Lukhele S, Nolte A (2021) | 7 | Proud to be a midwife | “Midwives get a lot of respect in my institution. I think it also depends on you, as a professional, how you carry yourself, how competent you are and you know you are empowered and upto- date … if you are empowered you get a lot of respect ...” (Participant 7, female, private) | U |  |  |
|  | 8 | Regulations and independent function | “In private hospital, patients belong to the obstetrician. Midwives hold the birth for the obstetrician. It’s a lot of frustration … we have to depend on the decision of [physicians].” (Participant 2, female, private) | U |  |  |
|  | 9 | Resource availability | “The way management treats the staff … instead of coming to support you, they are pressing you. When something goes wrong – instead of wanting to find out what went wrong they are judgmental, [rather] than helping you on how to make yourself better and how to improve.” (Participant 9, female, public) | U |  |  |
|  | 10 | Work burden | “Most of the time illegal immigrants do not book for antenatal care or place of delivery – but there is nothing we can do … we have to deal with it. You continuously take in patients whether the hospital is full or there is space.” (Participant 4, female, public) | U |  |  |
|  | 11 | Image of the midwife | “In the old days, [physicians] viewed midwives as the people that taught them. Some older physicians still talk about the “green epaulet girls” … the midwives who worked primarily in the district services. They viewed them as phenomenal … that they [midwives] knew what they were doing. Now they call [midwives] people who merely do the observations and don’t do them very well.” (Participant 1, female, public) | U |  |  |
| 4. Bradfield Z, Hauck Y, Kelly M, Duggan R (2019) a | 12 | Building relationships   - *Trusting relationship between woman and midwife* - *Relationship inclusive of partner and family* | “… you have that trust, a real trust in each other that was developed over time that you both rely on actually. She trusts you to make the right call and you trust her for her body to work efficiently and do what is necessary in the space that you’ve created” (P9).  “It [relationship] improves it [being with woman] because of the knowledge, because of knowing the family, knowing where they come from, know where they live, know what their background is … so that helps you antenatally but also for labour” (P7). | U |  |  |
|  | 13 | Woman centred care that is safe   - *Intuitive and individualised care founded in ‘knowing’* - *Addresses complexity of needs* - *Cultural safety* | “… it was just literally eye contact, me knowing that lady, knowing what she wanted, where she wanted to be … watching her body, watching what she was doing … I could pick up on that and jump in when it was relevant” (P4).  “… particularly if you’ve got women that have complex issues, you’ve got a full 4, 5 months of being with them to learn more about that problem rather than trying to be thrown in the deep end with someone in labour … you know their story, you know what they want, you get to spend more time with them, physically supporting them as opposed to have to sit there and read through their volume of notes to find out why does this woman not want this” (P5).  “I work a lot with Indigenous women, facilitating those relationships during pregnancy has made birthing so much more easier and … opens you up to the community because they know who you are and who you represent” (P3). | U |  |  |
|  | 14 | Impact on the midwife   - *Personal investment in professional practice* - *Enhances confidence* - *Recognition of importance to midwifery* | “… without knowing the women, it just wouldn’t be the same either so unfortunately it’s all or nothing from me … if I’m going to be around birth … I just can’t see it any other way” (P8).  “Wow that worked, ok we’ll do that next time … [if] that kind of situation presents I can do that again … So because I get the opportunity to practice I get … to see it work” (P2).  “… it’s important for midwives because lots of midwifery skills are being degraded or downgraded or lost” (P2). | U |  |  |
|  | 15 | Impact on the Woman  - *Reported benefits to women* | “That’s what you’ve done by being with woman … she will labour efficiently and she’ll feel so proud of herself afterwards to have done it under her own steam” (P9). | U |  |  |
|  | 16 | Challenges in the ‘known midwife’ model along with corresponding subthemes   - *‘Systems’ approach to childbearing* - *Midwives as the ‘vehicle’ for ‘with woman’* - *Women who are not known or don’t want to be ‘known’* | “… it’s [with woman] not supported in the majority of places where midwives work or models of care that midwives work in it’s not an acknowledged skill really. It’s not considered important, it’s not evidence based, it’s not scientific … it’s not supported and it’s not promoted as valuable” (P2).  “I think then there’s the scared of being told that they’re weird or there’s no science behind that, like being fobbed off because they do they make you feel unintelligent or because you believe in that [being with woman]” (P2).  “… it is different but … even if we haven’t met the woman specifically, we all know something about each other’s women … we’re all continuously talking so most of the time even if I haven’t met the woman I know something of her and something of her wishes” (P5). | U |  |  |
| 5. Bradfield Z, Hauck Y, Duggan R, Kelly M (2019) b | 17 | Essential to Professional Identity | “… is absolutely essential because without being ‘with woman’ you’re not a midwife, you’re just a person doing the job... that doesn’t actually provide true midwifery care” (KMP9). | U |  |  |
|  | 18 | Partnership   - Inclusive of support people | “ ... through your relationship with them you know that the woman trusts you, like a journey next to her, like a partnership [the woman] will listen to what you’re saying and taking it on board” (UMPKOP3). | U |  |  |
|  | 19 | Woman- Centred Practice  - Advanced communication skills  - Informed decision making  - Advocacy  - Adaptable  - Preparing the birth environment  - Midwifery presence  - Creating a space  - Personal qualities  - Therapeutic touch | “…listening to her birth plan, her ideas, her fears, getting that trust and you can almost have like a silent communication, you can just meet her eye and give the ‘it’s ok’ and she relaxes” (UMKOP11).  “…they’ve made a choice to come to this model of care and sometimes it ’ s a choice that you don’t always agree with ... but it’s the woman’s choice” (UMKOP7).  “You have to be the advocate and really speak up and [be] confident doing that.... because the woman sometimes says things to you afterwards and says thanks for speaking up for me”(UMKOP8)  “…based on where they ’ re at, how they ’ re coping, what they need and what they ’ re getting from the other people that are in the room... I go by their behav- iours, I go by what feedback they ’ re giving me, visual feedback like how they ’ re behaving ... they don ’ t say much to you in labour, then I go by what I do, how they respond to that … which might be them softening into whatever I ’ m doing” (KMP8).  “… trying to get them to find their own place in that horrible, stark, bright, noisy, non-natural birthing environment. So the bathroom ’ s a good place I try and make that environment as conducive to normal birth as possible. So dimming lights, having quiet conversations and just rubbing her back or holding her hand or just sitting with my hand on her belly kind of timing contractions and listening to her voice, how she ’ s coping with them” (UMP3).  “…being present in the moment and making yourself available and open to whatever it is that she re- quires or needs to help her through” (UMP9).  “… allows her body to work so the whole thing [labour] can move forward because you ’ ve just made this space … it ’ s definitely unique to midwifery because there ’ s no other situation where you ’ recreating the space that will facilitate the outcome” (UMKOP9).  “… respect is really import- ant and they [women] will receive that respect really well and likely mirror that and you get trust from that person through that, through showing respect” (UMP4).  “…being with woman you need to be able to touch her, to be able to calm her, massage, holding hands” (UMKOP6). | U  U |  |  |
| 6. Weltens M, Nooijer J, Nieuwenhuijze M (2019) | 20 | Knowledge | “What I find difficult… for example, prolonged rupture of membranes. The are obstetricians who say: if the client has 24 hours of rupture membranes around two/three o’clock at night, I would like to see her the evening before. And I think: Well that may also be the next morning. In this case, I often just wait until the next morning to call the obstetrician. Because if I make the call in the evening, then I know I need to refer them. In this, you indicate your own way of referring, and perhaps that is a little on experience, which allows you to deviate from the request of the obstetrician because you don’t see the need” (MS3) | U |  |  |
|  | 21 | Experience | “…[Trust] in normality of birth, but also in my own assessment, actually I am getting more experienced and I have seen more different cases. I have also seen the effects of my choices. More than in training, because in training you always have the backup of a skilled midwife. Now I am getting a level of confidence that tells me;… I dare to make this choice, I can do this “. (MJ2) | U |  |  |
|  | 22 | Physical and social environment | ‘And that I think makes it easier to refer, because you are already in the hospital. The referral is arranged easy and quick (MS1) | U |  |  |
|  | 23 | Influence of others | “I do think that we are really very afraid for everything. And that unrealistic fear leads to new thoughts. In particular the client, it seems that they always want the procedure available. […] I think that the client can have a strong voice, but only when they are well informed and not informed by the media [television, newspapers and magazines]. They are all so anxious”. (MS5) |  | C |  |
|  | 24 | Subjective factors | “We live in a society where we cannot deal with setbacks from nature anymore” | U |  |  |
| 7. Healy S, Humphreys E, Kennedy C (2017) | 25 | Professional autonomy and hierarchy in maternity care | “Unfortunately, our mums will have to be released by an obstetrician to come through the DOMINO scheme [midwifery-led program] at 20 weeks. I think it is totally unnecessary…. I think we are all capable of making our own decisions. So that´s just the way it is and we have to get it off the ground” [OLU (and MLU) midwife 4] | U |  |  |
|  | 26 | Midwifery-led care as an undervalued and unsupported aspiration | “It’s an unfortunate position that some DMOs [Designated Midwifery officers – who act liaison officers between the Health Service Executive and women seeking a homebirth], I don’t think, chose to be in that role and that’s a real disappointment because it could be a really, should be a really key role in developing an area [homebirth], in developing midwives and supporting them” [MLC midwife 5] | U |  |  |
|  | 27 | A shift in focus from striving for normality to risk management | “They are growing up in a medical environment…the students are learning from girls that came through this [medicalized] system as well, so it’s snowballing and what we used to have is slowly fading away” [OLU midwife 4] | U |  |  |
|  | 28 | Women view pregnancy through a ‘risk-lens’ | “I feel that the view out there is that the doctor knowns everything and the doctor is best and that they(women) believe the obstetrician” (OLU midwife2) | U |  |  |
| 8. Amiri-Farahani L, Gharacheh M, Sadeghzadeh N, Peyravi H, Pezaro S (2022) | 29 | Emphasis on the  non‑interventional care | “When it comes to childbirth and how a midwife supports a woman in labour, I think it’s my job to be able to manage a good labour. Midwifery is not just being able to pull the baby out, but to provide psychological and physical support for woman in labour and to allow childbirth to be progress with less intervention and successfully managing the woman in labour.” | U |  |  |
|  | 30 | Midwifery-specific focus | “A sense of conscience [which] compels me to take care of the patient according to standard and basic principles. I try to do my job properly even when there is no  supervision of an obstetrician.” (Maryam) | U |  |  |
|  | 31 | Obstacles and Challenges of midwifery care   - Lack of familiarity with and knowledge in relation to childbirth - The insignificant role of midwife in decision making - Obstetrician utilitarianism - High workload along with work-related stress - An absence of a triangle of trust in care | “We had a patient last night who had dilation of 7-8 cm and she was progressing well. Then the patient deteriorated and her O2 saturation dropped, so that we had to administer some oxygen, and obstetrician took her for cesarean section. We could not comment, because we have no role in decision making.”  “I feel like I’m a robot in providing care. I feel like midwifery is a dependent profession. In a way, we have to do flattery to someone. Obstetrician gives order and we have to implement it, then the obstetrician comes and blames us for not doing certain things; I kind of feel that I am a robot.”  “If patient has dilation of 6-7 cm, we have to call the obstetrician to come and perform the delivery. Usually, because of financial issues, the obstetricians perform all deliveries because they do not want to share their income. If they are very desperate, they will ask us to perform the delivery, but they administer the epidural in order not to give us too much money” (Zahra)  “It disappoints me when I do my best to take care of a patient, but I see that the doctor does his best to perform delivery by herself and does not treat me as a person who has been there for hours taking care of patient”  “…our workload has increased so much. Now every midwife is responsible for at least three patients. The midwife in charge of newborn must be next to mother to teach her how to breastfeed. Finally, no matter how good we are, we cannot provide good psychological support for patients during labour” (Fatemeh) | U  U |  |  |
| 9. Ferguson B, Baldwin A, Henderson A, Harvey C (2022) | 32 | Perceptions and behaviours   - Behaviours guided by woman centred care - Behaviours guided by protocols | “It gives you confidence and validation in communicating to medical staff. It ’ s got more guts behind it, especially when you ’ re junior and escalating. You ’re recovering your bum and they ’ re aware that if something happens later, you will say I told you so!” (Jane)  “The EWT protocol dictates that I must tell doctors about it, and they must review. It’s a great help.” (Andrea) | U |  |  |
|  | 33 | Shifting practice   - Compliance - Defiance | “Midwives tailor their care to the woman individually to a degree. We’re still told we’ve got to do these observations in the mandated timeframe, but there are ways around it, like choosing when to take the obs and who to escalate to” (Parvin)  “My registration is on the line and the hospital policies are telling me what to do. It is a line in the sand, and for the sake of your registration, you must come to the line” (Brooke)  “We cannot go away from observation charts, and as a home birth midwife, I use an old Partogram. I write on my Partogram the labour progress so that if I ’ m audited, I can show that I ’ m safe. | U |  |  |
|  | 34 | Power within practice   - Accountability of care provision - Autonomy - Tension across accountability and autonomy | “I was spending a lot of time being cranky and that was just the system, it wasn’t good for my soul. When I left it was frustration at working in a system that wasn’t looking out for women”. (Millicent)  “We are dictated to that we must take observations at specific times, that this labour must fit within these timeframes. It’s ridiculous, it takes away your autonomy, your ability to make judgments for yourself.” (Parvin)  “It’s all about the mighty dollar, a woman is allowed to occupy this bed for a certain amount of time because more women are coming, and you don’t have the bed capacity or staff to look after long labors”. (Misako) | U |  |  |
| 10. Spendlove Z (2018) | 35 | Anxiety over uncertainty, error and subsequent blame provoking defensive practice | “We’re in that society, that culture at the moment where somebody is always looking to blame…there’s always a scapegoat… and it’s sad that it’s like that…risk is dictating everything we do in maternityt”(Sara, midwife for 12 years) | U |  |  |
|  | 36 | Tension of risk work: risk minimisation versus risk causation | “Risk management is actually stopping us caring. You know, I’ve seen it first-hand where a newly qualified midwife was concentrating so much on filling her paperwork in that she forgot there’s a woman there she needs to care for”. (Sue, midwife for 10 years) | U |  |  |
|  | 37 | Shifting of professional role boundaries | “We’ve been sucked into a biomedical model of care, we’re frightened to practice any other way. Autonomy has gone…we are fed by CNST [Clinical Negligence Scheme for Trusts] We’re fed by Trust policies. We are like a conveyor belt of semi-professionals…we’re just like clockwork toys. We have to just do as we’re told really. And we’re scared to go against that, just in case it [care] goes wrong. As then they’re going to say, well, the guideline says you should’ve done this and you didn’t do that”. (Jenny, midwife for 18 years) | U |  |  |
| 11. Zondag L, Maas V, Beuckens A, Nieuwenhuijze M (2022) | 38 | Attitudes Toward Interventions | “Everything [that] influences the natural behavior of the woman giving birth … In case a woman needs some encouragement, such as ‘you can do this, it’s going well.’ This is also an intervention for me.” (Midwife 12) | U |  |  |
|  | 39 | Influences on Midwives’ Attitudes:   - *Experiences in Collaboration* - *Trust and Fear* - *Woman-Centeredness* | “I sometimes think: ‘what does the obstetrician think about it [care management]?’. That is on my mind when I don’t achieve a certain result within a certain time.” (Midwife 7)  “I certainly feared upright births in my early years. After the baby was born, the blood clattered down and I wanted to administer oxytocin right away. After a while, you get used to the noise and you can estimate the blood loss better, and you hold off administering oxytocin”. (Midwife 14)  “We explain [in the pregnancy]: ‘… if you have a lot of blood loss we will administer oxytocin.’ They will agree to it. During birth, you also need to obtain informed consent. I find that difficult. Sometimes I just want to administer it”. (Midwife 15) | U |  |  |
|  | 40 | Supportive and Directive Styles | “two attitudes emerged that influenced midwives’ approach toward perinatal care: a wait and see attitude and a check and control attitude. In the 3 subthemes, the  midwives in our study described what experiences, beliefs, and values influenced their attitudes” | U |  |  |
| 12. Nilsson C, Olafsdottir O, Lundgren I, Berg M, Dellenborg L (2019) | 41 | The birthing rooms and the office: Different rooms of care | “The atmosphere in the birthing room during one observation was very intense, but still calm and filled with trust. The birthing woman was sitting like a queen in her bed; she had strong contractions and inhaled Entonox with silent breaths. Her mother and sister were there, holding her hands. The midwife was present at her side, occasionally listening to the baby’s heart by using a Pinard. She later described the ward as being very hectic that day, but in this room it was calm. When the researcher informed the woman that the study concerned midwives’ care, she said with a big smile that her midwife was wonderful: ‘You must pass her with distinction. She was breathing with me, and that made me calm”. | U |  |  |
|  | 42 | Women giving birth or being delivered: Midwives’ expectations and relationships with women | “It’s so nice to see a primipara who doesn’t demand an epidural”. | U |  |  |
|  | 43 | Old and new caring roles of the midwife: Women giving birth in a “new age” | “One day, the following was observed at the labour ward: A woman in advanced labour who was recently admitted told her midwife that she wanted an epidural. The midwife asked her to reconsider and explained that the preparations would take much of her time, with the risk of leaving the woman alone without a midwife being at her side. Despite the midwife’s concerns, the woman chose the epidural. However, when all the preparations were completed she started to push and gave birth just a few minutes later. The midwife explained later to the ethnographer that she wanted to be honest with the woman and make sure she understood the possible consequences of her choice. The midwife’s interpretation was that the woman did not dare to give birth without the pain relief, while from her experience she knew that she as a midwife had the possibility to support and induce courage with her presence”. | U |  |  |
|  | 44 | Being and doing: Different approaches to caring | “Actually, most women know how to give birth, you only have to reassure them in what they are doing” (…) ‘only being’ with a woman” | U |  |  |
|  | 45 | Holistic and reductionist care: Guided by contrasting models and guidelines | “According to the guidelines we should… A midwife and a physician had a discussion in the office concerning which method to use for inducing a woman’s labour. The midwife suggested a method having the drug already prepared in her hand. The physician instead pondered on different suggestions from colleague physicians. The discussion ended with the physician saying: ‘I will examine the patient and discuss the case with my colleague, and then I let you know what to do’. Despite the determined tone, the physician seemed unsure about the midwife’s suggestion and wanted to consult with a colleague, excluding the midwife from this talk. The midwives in turn tended to exclude physicians because they preferred to consult with each other before consulting the physicians. They felt that the morning rounds with physicians focused on the medical and organizational aspects of childbirth to the exclusion of midwives’ views on the normality of childbirth and caring issues. The midwives therefore discussed the need for a midwife-round before the ordinary round with the physicians where they could consider more midwifery caring issues and not just the medical and institutional aspects of childbirth as they usually did during the morning rounds”. | U |  |  |
| 13. Aanensen E, Skjoldal K, Sommerseth E, Dahl B (2018) | 46 | How midwives’ perceptions of their own as well as the women’s personal attributes and attitudes influence the birthing process:   - Some women have it in them to give birth normally - Some midwives have the attributes to promote a normal birth | “I believe that the laboring woman’s knowledge and her attitude toward being pregnant and giving birth is essential. If the woman is anxious and has absolutely no faith in her ability to give birth, the starting point for achieving a normal birth is lacking”. (2)  “I think that promoting a normal birth is connected with your confidence as a midwife. When you are experienced and confident, you dare to look away from the CTG-printout and all the things that distance you from what is normal”. (3) | U |  |  |
|  | 47 | Lack of time and impatient staff negatively affect the woman’s chances of giving birth normally:   - Busy shifts reduce midwives’ possibility of being present for the woman and promoting a normal birth - Impatient midwives and doctors deprive the woman of her possibility to give birth normally | “Of course, if a laboring woman really needs me and the ward is busy, it is easier to give her an epidural, because then she may be able to cope with labor without me  being continuously present”. (7)  “It seems that midwives and obstetricians find it handy to kind of “hand the woman her baby.” Thanks to our effort, the woman gives birth. I think the woman believes it is like that. Sort of, ‘thank you for having saved me.’ But it is really the woman herself who has given birth”. (3) | U |  |  |
|  | 48 | Increasing use of procedures, selection criteria, and technology threatens midwifery skills and prevents midwives from promoting normal births:   - Midwives feel monitored and controlled and fear they are becoming obstetric nurses - Selection criteria and procedures restrict the possibility for women to give birth normally - Midwifery skills must give way to the use of technology | “The doctor on night shift should be able to sleep safely, trusting that the midwife knows how to handle a normal birth and that she will call him should complications occur. Today, the doctors are breathing down my neck, monitoring my actions and disturbing my work, creating an insecure environment for the laboring woman. I miss doctors and midwives who are confident in their work”. (5)  “I keep thinking, am I following the procedure when I do this? Sometimes, I am more  concerned about following the procedure than considering what is best for the woman”. (8)  “It does something to you, having a needle put in your back… An epidural pump and all the intravenous equipment. Use of STAN. All this equipment is pushed, pulled, and connected to a socket. Cables often intertwine… That scenario is unlikely to create a sense of being empowered”. (3) | U |  |  |
| 14. Oliveira J, Correia Campo T, Lima Costa Souza F, Davim R, Costa Dantas, J (2016) | 49 | Atividades Assistenciais | “[...] A gente faz triagem, acolhimento, classificação de risco, admissão, partograma, exame obstétrico, medidas não farmacológicas para alivio da dor, orientações sobre período expulsivo, aleitamento, cuidados com o recém nascido, conduz o parto sem distócias de maneira humanizada, puerpério, partos de ofiu, indução com prescrição médica (misoprostol), cardiotocografia, proteinúria, HGT, cuidados e assistência com recém-nascido quando não tem o pediatra, além de registros e sistema informatizado, checklist, material do setor”. (E5) | U |  |  |
|  | 50 | Educação em Saúde | “[...] Sinto a angústia das parturientes para que se sintam bem [...] e, na hora do parto passar tranquilidade, menos manobras perineais, sem pressa”. (E9) |  | C |  |
|  | 51 | Apoio da direção institucional | “[...] a gente tem total apoio da gerência do hospital, autonomia e diferentemente de outras instituições aqui o enfermeiro obstetra conduz o parto, tem empoderamento de qualidade e confiança”. (E2) | U |  |  |
|  | 52 | Trabalho em equipe multiprofissional | “[...] quando se trabalha em equipe multiprofissional e todos falam a mesma língua é mais fácil chegar ao êxito”. (E7) | U |  |  |
|  | 53 | Pouco conhecimento das parturients em relação às fases do trabalho | “[...] Com a parturiente despreparada para o parto nosso trabalho se torna mais difícil, é necessária muita paciência, explicações e estar mais junto à mulher e família”. (E1) | U |  |  |
|  | 54 | Pouco tempo de experiência com assistência à parturiente | “[...] como estou há pouco tempo atuando tenho dúvidas e muita insegurança”. (E2) | U |  |  |
| 15. Armanmehr V, Banafshe E, Hashemizadeh H, Alami A, Khajavi (2023) | 55 | Responsive staff   - Up-to-date knowledge - Essential skills - Attitude towards natural childbirth | “If we want the program to achieve its goals, midwives and obstetricians should participate in retraining courses, so they will be less resistant to changes.” (P4)  “Relying only on academic training, physicians and midwives won’t get the necessary preparation for giving birth. That’s why most of them are panic when they enter the delivery room and order a cesarean section because they don’t have enough skills and experience.” (P8)  “Many obstetricians do not believe in natural childbirth, and this is transferred to the parturient. Many of the mothers wonder whether their doctor has given birth through vaginal delivery or not. They want to take example from them.” (P13) |  | C |  |
|  | 56 | Characteristics of the parturiente   - Belief in own ability - The right to choose the mode of delivery - Socio-economic status | “Today’s women do not believe in their own ability to give birth naturally. They often think they should give birth under the 100% care of a doctor and medical technology.” (P16)  “If the mother herself chooses a natural birth, she will more easily accept the pain and stress caused by the natural birth process and will be less impatient; as a result, there will be higher chances for a natural birth.” (P14)  “A woman who is literate and to whom we give educational materials (videos, books, etc.) welcomes the natural childbirth and cooperates better during childbirth.” (P1). |  | C |  |
|  | 57 | Recognition of midwifery role   - Transparency of duties and rewards - Professional independence | “The lack of clarity in some of the job descriptions and regulations in the delivery ward may be the only factor that prevents this program”, said one of the midwives (P8).  “Based on this instruction, midwives’ work is gradually being taken away... at the moment of delivery, the doctor comes and performs the delivery,…this is the most difficult thing for me (tears welling up in her eyes)...the midwife wants to deliver the baby by herself.” (P2) | U |  |  |
|  | 58 | Teamwork   - A sense of shared identity - Goal-oriented interactions | “Everyone should consider himself/herself as ‘we’, so that natural childbirth can be promoted.” (P13)  “Instead of looking at it like a supervisor–subordinate relationship, doctors and midwives should benefit from each other’s work experience and pursue the health of the mother and baby as a single goal,...but this is not the case.” (P6) | U |  |  |
|  | 59 | The birthing environment   - Necessary equipment - The physical environment - The private environment | “Any goal is achievable with a series of tools. For a natural delivery, the presence of equipment such as aromatherapy, heat and cold therapy, etc. is necessary to give confidence to the delivery team and encourage  the cooperation of the parturient.” (P7)  “If the environment of the maternity hospital becomes too homely and the equipment is not seen, the mother may start to worry and think that we do not have access to the necessary equipment.” (P13)  “If implemented correctly, the LDR (Labor, Delivery, Recovery) system will let the mother wear her favorite clothes during hospitalization and listen to her favorite music. The stages of labor can be done in a private room next to her husband or her favorite companion. This plays a very important role in making natural childbirth a pleasant experience and promoting it.” (P9) |  | C |  |
|  | 60 | Effective management   - Manager selection system - Performance monitoring and evaluation | “The deputy of treatment was changed several times, followed by changing the head of the hospital…these rapid changes prevent the continuous and uniform implementation of the program.” (P6)  “statistics should be checked by the management; they should find out the problems, and see what percentage of cesarean sections could have been normal, so that things can be corrected.” (P10) | U |  |  |
|  | 61 | Institutional and social context   - The payment system - Social values | “The fact that a financial incentive is included in this program has increased the motivation of midwives to attend natural childbirth.” (P2).  “The taboos associated with natural birth must be eliminated in society; there are many misconceptions people have about it, or they think the pain is unbearable.” (P9) |  | C |  |
|  | 62 | Social education   - Inclusive and continuous education - Effective education | “Everyone should be trained, from managers…to even the hospital guards, all of them can help us. For example, if a high school principal comes here and is educated, after she gives birth, this will also affect both her students and her colleagues, and thus the whole society is educated.” (P6)  “The more the trainer can gain the mother’s trust…, the more likely her attitude will change, and natural birth will be her first choice.” (P5) | U |  |  |
| 16. Ferreira Júnior A, Brandão L, Teixeira A, Cardoso A (2021) | 63 | Conhecimento e autonomia | “Mas tem muita coisa aqui, é um campo riquíssimo, é muito prazeroso, é muito bom, a gente tem muita autonomia, mas tem a questão de ter muita responsabilidade também”. (E6) | U |  |  |
|  | 64 | Credencialismo | “Aqui não tem médico, aqui são só os enfermeiros e os técnicos. O médico, nós chamamos quando julgamos necessário, quando tem alguma intercorrência. Aqui somos nós que fazemos os partos”. (E4) | U |  |  |
|  | 65 | Divisão do trabalho | “O trabalho burocrático, às vezes, dificulta a assistência. Porque partejar não é fácil, precisa coragem, paciência, tempo [...] porque é muito fácil você acelerar um parto”. (E5) |  | C |  |
|  | 66 | Mercado de trabalho | “O CPN foi uma nova oportunidade de trabalho, que agregou o que eu já fazia em centro obstétrico, mas com maiores responsabilidades, porque agora nós somos responsáveis por mais coisas. E o jeito de fazer mudou, porque a enfermagem deixa a mulher mais livre também”. (E5) | U |  |  |
|  | 67 | Quadro de Valores | “O CPN trouxe mais autonomia para a enfermeira, porque nos deixa mais livres para desenvolver nosso trabalho. Ainda há dificuldades que não foram superadas. Posso citar a assinatura da Autorização de Internação Hospitalar que não foi liberada ainda para que a gente faça aqui. Tem regulamentação para isso, mas não tem sido implantado nos CPN”. (E3) | U |  |  |
