# Supplementary material for: Midwives’ and Nurse Midwives’ Autonomy Experience in Caring for Laboring Women in Health Facilities: A Systematic Review With Metasynthesis
Source: SAGE Open Nurs. 2026 Sep 7;12:23779608261433120. doi: 10.1177/23779608261433120 (PMC13554601; doi:10.1177/23779608261433120)
Supplement: Supplementary file 4 [file sj-docx-4-son-10.1177_23779608261433120.docx]

**Appendix IV. Aggregation process for primary data**

| **Example** | **Findings** | **Subcategory** | **Category** | **Meta-category** |
| --- | --- | --- | --- | --- |
| ‘The first thing I think, which I always have in the back of my mind, is what can I as a midwife do to make this woman feel safe and well cared for? How can I ensure that she can be herself when she is giving birth to her child, and that I can give her, to the greatest extent possible, what she needs and wants? I have to “tune in” to this woman, to the woman in labor.’(Remvik-Larsen et al., 2023) | 1(U), 9 (U), 10 (U), 22(U), 35 (U), 36 (U), 37 (U), 49 (U), 58 (U), 60 (U) | Establishing a trusted environment (U) | **Autonomy as a context of safety and confidence** | **The dynamics of Midwives/Nurse Midwives’ autonomy** |
| [...] a gente tem total apoio da gerência do hospital, autonomia e diferentemente de outras instituições aqui o enfermeiro obstetra conduz o parto, tem empoderamento de qualidade e confiança.(Oliveira, 2016) | 8 (U), 25 (U), 26 (U), 27 (U), 28 (U), 33 (U), 47 (U), 48 (U), 51 (U), 52 (U), 53 (U), 54 (U) | Recognizing institutional support (U) |  |  |
| “I feel like I’m a robot in providing care. I feel like midwifery is a dependent profession. In a way, we have to do flattery to someone. Obstetrician gives order and we have to implement it, then the obstetrician comes and blames us for not doing certain things; I kind of feel that I am a robot.”(Amiri-Farahani et al., 2022) | 31 (U),61 (C),62 (U), 64 (U),65 (C), 66 (U) | Dealing with challenges for M/NM (C) |  |  |
| ‘I hear how people breathe, and I hear when their contractions start and stop, and I see how the women move, so that's my most important instrument. I don't have to listen so much to fetal sounds when I know she has good breaks between contractions. Because that tells me that the baby is getting oxygen in between times.’(Remvik-Larsen et al., 2023) | 2 (U), 14 (U), 16 (U), 17 (U), 30 (U), 34 (U), 38 (U), 39 (U), 57 (U), 67 (U) | Being and feeling M/NM (U) | **Autonomy as a process of strengthening**  **identity** |  |
| ‘… it was just literally eye contact, me knowing that lady, knowing what she wanted, where she wanted to be … watching her body, watching what she was doing … I could pick up on that and jump in when it was relevant.’ (Bradfield et al., 2019) | 12 (U), 13 (U), 15 (U), 18 (U), 19 (U), 29 (U), 32 (U), 40 (U), 50 (C), 56 (C) | Caring for women in their uniqueness (C) |  |  |
| ‘During childbirth I try to say to myself: “hold back, you don't have to [artificial rupture the membranes]. The woman does not benefit from that. She will only have more severe contractions. That's of no benefit”.’(D.Zondag et al., 2022) | 4 (U), 5 (U), 6 (U), 20 (U),21 (U), 23 (C), 24 (U), 46 (U), 55 (C), 63 (U) | Improving critical thinking (C) | **Autonomy as a reflection of expertise** |  |
| ‘The longer you work, the easier it becomes to feel free in decision-making and to decide what is best in each situation…(D.Zondag et al., 2022) | 3 (U), 7 (U), 11 (U), 41 (U), 42 (U), 43 (U), 44 (U), 45 (U), 59 (U) | Clinical practice enhaces the primacy of care (U) |  |  |
| (U) Unequivocal |  |  |  |  |
