# Supplementary material for: Midwives’ and Nurse Midwives’ Autonomy Experience in Caring for Laboring Women in Health Facilities: A Systematic Review With Metasynthesis
Source: SAGE Open Nurs. 2026 Sep 7;12:23779608261433120. doi: 10.1177/23779608261433120 (PMC13554601; doi:10.1177/23779608261433120)
Supplement: Supplementary file 5 [file sj-docx-5-son-10.1177_23779608261433120.docx]

**Appendix V. ConQual summary of findings**

| **Systematic review title: Midwives’ and Nurse Midwives' autonomy experience in caring for laboring women in health facilities: A Systematic Review with Metasynthesis** | | | | |
| --- | --- | --- | --- | --- |
| **Population:**  Midwives and Nurse Midwives  **Phenomena of interest:** experiences and perspectives of autonomy from midwives and nurse midwives  **Context:**  In health facilities that provides care to women in labor | | | | |
| **Synthesised finding** | **Type of research** | **Dependability** | **Credibility** | **ConQual score** |
| Autonomy as a context of safety and confidence | Qualitative | Higth | Moderate | Moderate |
| Autonomy as a process of strengthening identity | Qualitative | Higth | Moderate | Moderate |
| Autonomy as a reflection of expertise | Qualitative | Higth | Moderate | Moderate |
